# A Tissue Engineered Blood Vessel Model of Hutchinson-Gilford Progeria Syndrome Using Human iPSC-derived Smooth Muscle Cells

**DOI:** 10.1038/s41598-017-08632-4

**Published:** 2017-08-15

**Authors:** Leigh Atchison, Haoyue Zhang, Kan Cao, George A. Truskey

**Affiliations:** 1 Department of Biomedical Engineering at Duke University, Durham, NC 27708 United States; 2 Department of Cell Biology and Molecular Genetics at University of Maryland, College Park, MD 20742 United States

## Abstract

Hutchison-Gilford Progeria Syndrome (HGPS) is a rare, accelerated aging disorder caused by nuclear accumulation of progerin, an altered form of the Lamin A gene. The primary cause of death is cardiovascular disease at about 14 years. Loss and dysfunction of smooth muscle cells (SMCs) in the vasculature may cause defects associated with HGPS. Due to limitations of 2D cell culture and mouse models, there is a need to develop improved models to discover novel therapeutics. To address this need, we produced a functional three-dimensional model of HGPS that replicates an arteriole-scale tissue engineered blood vessel (TEBV) using induced pluripotent stem cell (iPSC)-derived SMCs from an HGPS patient. To isolate the effect of the HGPS iSMCs, the endothelial layer consisted of human cord blood-derived endothelial progenitor cells (hCB-EPCs) from a separate, healthy donor. TEBVs fabricated from HGPS iSMCs and hCB-EPCs show reduced vasoactivity, increased medial wall thickness, increased calcification and apoptosis relative to TEBVs fabricated from normal iSMCs or primary MSCs. Additionally, treatment of HGPS TEBVs with the proposed therapeutic Everolimus, increases HGPS TEBV vasoactivity and increases iSMC differentiation in the TEBVs. These results show the ability of this iPSC-derived TEBV to reproduce key features of HGPS and respond to drugs.

## Introduction

HGPS is a rare genetic disease caused by a single point mutation in the Lamin A/C (*LMNA*) gene. The disease is characterized by accelerated aging in affected children due to the nuclear accumulation of a truncated and farnesylated form of prelamin A called progerin^1^. Due to the retention of this farnesyl group, progerin toxicity is based on its strong binding to the nuclear membrane which causes abnormal nuclear membrane shape^2^. This disruption of nuclear structure subsequently leads to a variety of downstream defects in DNA repair, proliferation, and accelerated cell senescence, all of which are associated with nuclear function^3^. The progerin protein primarily affects cells of the mesenchymal lineage including vascular smooth muscle cells (SMCs)^4^. At present, no cure exists for HGPS and the ultimate cause of death is atherosclerosis or stroke at 10-15 years, possibly or at least partially due to smooth muscle cell senescence and loss in the cardiovascular system^5, 6^. Moreover, the vasculature of HGPS patients presents excessive calcification, lipid accumulation, vessel wall thickening and fibrosis due to SMC malfunction^7^.

The presence of progerin in older, non-HGPS individuals is thought to be due to the sporadic use of the same cryptic splice site in the *LMNA* gene that is constitutively active in HGPS^8^. The discovery that progerin concentration increases in an age-dependent manner and causes many of the same cellular and cardiovascular phenotypes associated with human aging, has sparked interest in studying HGPS in order to better understand the normal aging process^9^. Treatment of HGPS may ultimately help determine therapeutic targets to reduce the effects of aging^10^.

A factor limiting advances in the field is that HGPS disease progression and drug effects are primarily studied in 2D cell cultures or rodent models due to the limited number of autopsy specimens and human patients available^11–13^. Although 2D iPSCs and mouse models provide a useful screen for drug therapies and disease development, they do not fully or accurately depict the human disease state in arteries, complicating efforts to make definite conclusions on the correlation between HGPS and normal age-related cardiovascular disease^14^.

An *in vitro* 3D tissue model using human cells that incorporates a physiologically relevant biomechanical environment can provide a better representation of the disease phenotype compared to 2D tissue culture^15^. In addition, 3D culture systems containing multiple vessel wall cell types have the capability of examining functional responses analogous to those performed clinically^16^. Since the primary cause of death for HGPS patients is cardiovascular disease, a 3D tissue engineered blood vessel (TEBV) model that mimics the basic organization of human vasculature enables a better understanding of the link between HGPS and normal cardiovascular aging. It also has the potential to act as a safe, inexpensive and effective test bed for therapeutics that could aid not only HGPS patients, but the general population at risk for age-related cardiovascular disease.

Current efforts to fabricate 3D vascular constructs to study various cardiovascular diseases have focused on deriving large numbers of the two main cell types responsible for vessel function, SMCs and endothelial cells (ECs), both of which are involved in many vascular diseases. Many of these studies have used animal cells due to the difficulty in obtaining human sources as well as to avoid the need for immunosuppression in immunocompetent animal models^17^. Human iPSCs are an attractive source for these vascular cell types due to the ability to easily expand and culture iPSCs prior to differentiation to the desired cell type as well as the ease of acquisition from human subjects. In terms of SMCs, this is particularly important due to the slow culture growth and quick senescence of primary cell sources^18^. iPSCs also provide the ability to create patient specific disease models due to their capability to maintain a disease phenotype post-differentiation^12^. This is useful for rare genetic disorders such as HGPS where the donor pool is limited. By validating a TEBV disease model of HGPS using iPS-derived cell sources, a variety of rare genetic disorders associated with the cardiovascular system can be studied. This model also provides a better *in vitro* platform for comparing normal human cardiovascular aging and HGPS for future therapeutic discoveries.

In this study, we investigated the function of TEBVs using SMCs differentiated from iPSCs (iSMCs) derived from a previously well-characterized healthy and HGPS donor in TEBV constructs^19^. We fabricated these TEBVs with either normal or HGPS iSMCs in the medial wall and human cord-blood endothelial progenitor cells (hCB-ECs) from a separate donor in the lumen, allowing us to isolate and study the effects of the two iSMC sources on TEBV structure and function. The iSMCs show stable function within these TEBV constructs in response to known cardiovascular stimulants over multiple weeks. Additionally, TEBVs fabricated from iSMCs derived from HGPS donor cells develop pathologies associated with the HGPS cardiovascular phenotype. Furthermore, we can ameliorate the reduced vasoactivity seen in HGPS iSMC TEBVs through short term treatment with the rapamycin analog, RAD001 (Everolimus).

## Results

### Functional Characterization of iSMC TEBVs in Response to Vasoagonists

In order to establish the utility of using SMCs derived from iPSCs, we first investigated the function of TEBVs fabricated from iSMCs in our *in vitro* perfusion system as previously described^20^. Briefly, fibroblasts from a healthy donor (HGFDFN168) and a HGPS patient (HGADFN167 with the 1824 C > T substitution at exon 11) were converted to iPSCs and then differentiated into iSMCs using a previously defined 31 day differentiation protocol^21^. Immunofluorescent staining of HGPS and normal iSMCs in 2D culture, prior to formation of TEBVs, shows that these iSMCs were present at high purity and expressed abundant alpha smooth muscle actin and calponin (Fig. S2D). Few cells were positive for smooth muscle myosin heavy chain, as previously reported^21^.

**Figure 1:**
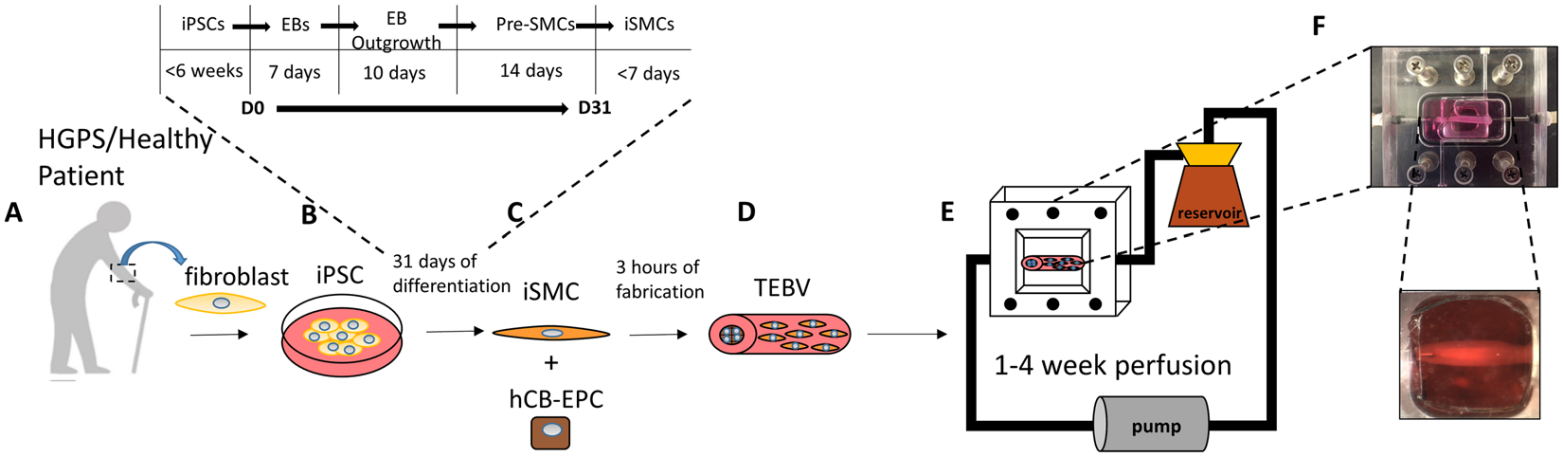
Schematic diagram of the procedure to produce iPSC-derived SMC TEBVs from healthy and HGPS patients. (**A**) A fibroblast biopsy from either healthy (young or old) or HGPS individuals (**B**) is converted to induced pluripotent stem cell (iPSC) cultures. (**C**) iPSCs are then differentiated into induced smooth muscle cells (iSMCs) using a 31-day process as previously described by Xie *et al*.^21^. (**D**) iSMCs are then incorporated into a dense collagen gel construct that is seeded with human cord blood-derived endothelial cells from a separate, healthy donor on the luminal surface to create iSMC TEBVs using the process previously described by Fernandez *et al*.^20^. (**E**) iSMC TEBVs are then incorporated into a flow loop and perfused with steady laminar flow at a shear stress of 6.8 dynes/cm^2^ for 1 to 4 weeks for maturation and functional characterization studies. (**F**) Photographic images of HGPS iSMC TEBVs in the custom perfusion chambers under perfusion conditions.

**Figure 2:**
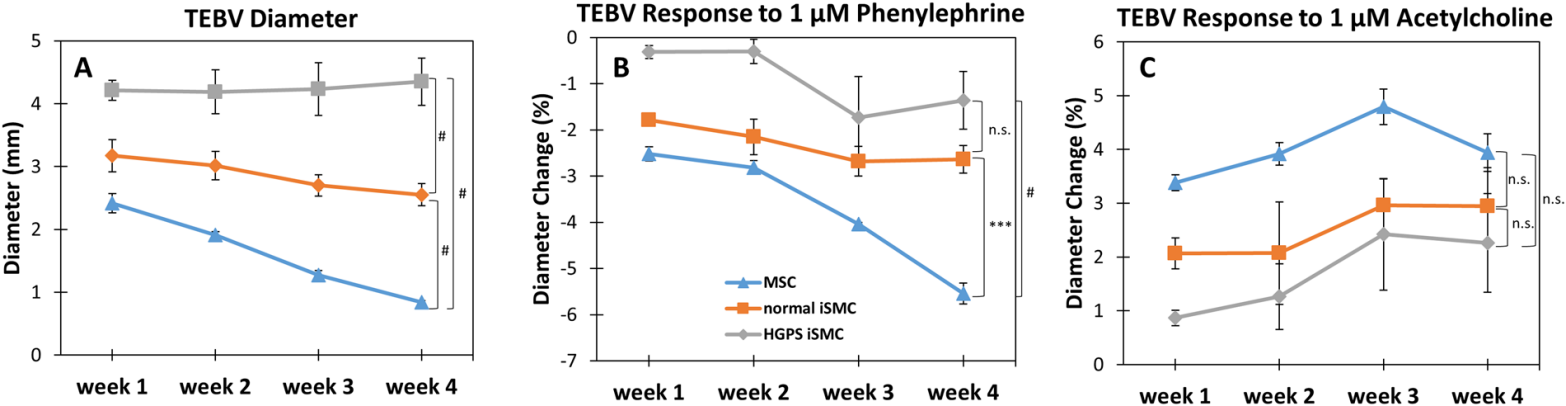
Functional characterization of MSC or iPSC-derived SMC TEBVs from healthy and Progeria patients. (**A**) Weekly outer diameter measurements of TEBVs fabricated with either MSCs, normal iSMCs, or HGPS iSMCs and seeded with hCB-ECs. (**B**) Weekly response to 1 μM phenylephrine of TEBVs fabricated with either MSCs, normal iSMCs, or HGPS iSMCs and seeded with hCB-ECs. (**C**) Weekly response to 1 μM acetylcholine of TEBVs fabricated with either MSCs, normal iSMCs, or HGPS iSMCs and seeded with hCB-ECs. Data are represented as mean ± S.E.M. n = 3 TEBVs. n.s. = not significant, ***P < 0.001, ^#^P < 0.0001 at week 4.

TEBVs were fabricated with either human mesenchymal stem cells (MSCs), normal iSMCs, or HGPS iSMCs in the medial wall and seeded with hCB-ECs in the lumen^20^. These hCB-ECs function the same as vessel wall derived ECs, allowing us to isolate and identify effects induced by HGPS-derived SMCs in TEBVs^22^. Within two hours of preparation, the TEBVs were integrated into a continuous laminar flow loop running at 2 ml/min to simulate a physiological shear stress of 6.8 dynes/cm^2^ (Fig. 1). We ran perfusion experiments for multiple weeks and assessed the vasoactivity of iSMC TEBVs compared to TEBVs fabricated from MSCs on a weekly basis (Fig. 1). MSCs were chosen as the control primary cell source for comparison of functionality due to their ability to terminally differentiate into the smooth muscle lineage^23^. TEBVs fabricated with iSMCs showed overall larger diameters that did not show any statistically significant decline in diameter over time due to differentiation towards a contractile SMC phenotype, unlike MSC TEBVs as we previously observed (Fig. 2A)^20^. MSC TEBVs also showed a significant increase in vasoconstriction in response to 1 μM phenylephrine over four weeks unlike iSMCs TEBVs whose response changed by a much smaller amount over this time period (Fig. 2B). All conditions showed a trend in which vasodilation in response to 1 μM acetylcholine over four weeks increased with perfusion time (Fig. 2C). This vasoactive response, however, was reduced in iSMC TEBVs compared to MSC TEBVs; most significantly vasoconstriction in response to 1 μM phenylephrine at week four was much less than that observed with MSC TEBVs. Initially at week one, however, vasoactivity and diameters are not significantly different between normal iSMC TEBVs and MSC TEBVs, further indicating the ability of MSC TEBVs to continuously mature to a very contractile phenotype over time. TEBVs fabricated from iSMCs of HGPS patients showed the most reduced vasoactivity and the largest overall diameters in comparison to normal iSMC and MSC TEBVs at all time points, but this reduced response is only significantly different at earlier time points.

### Phenotypic Characterization of iSMC TEBVs

**Figure 3:**
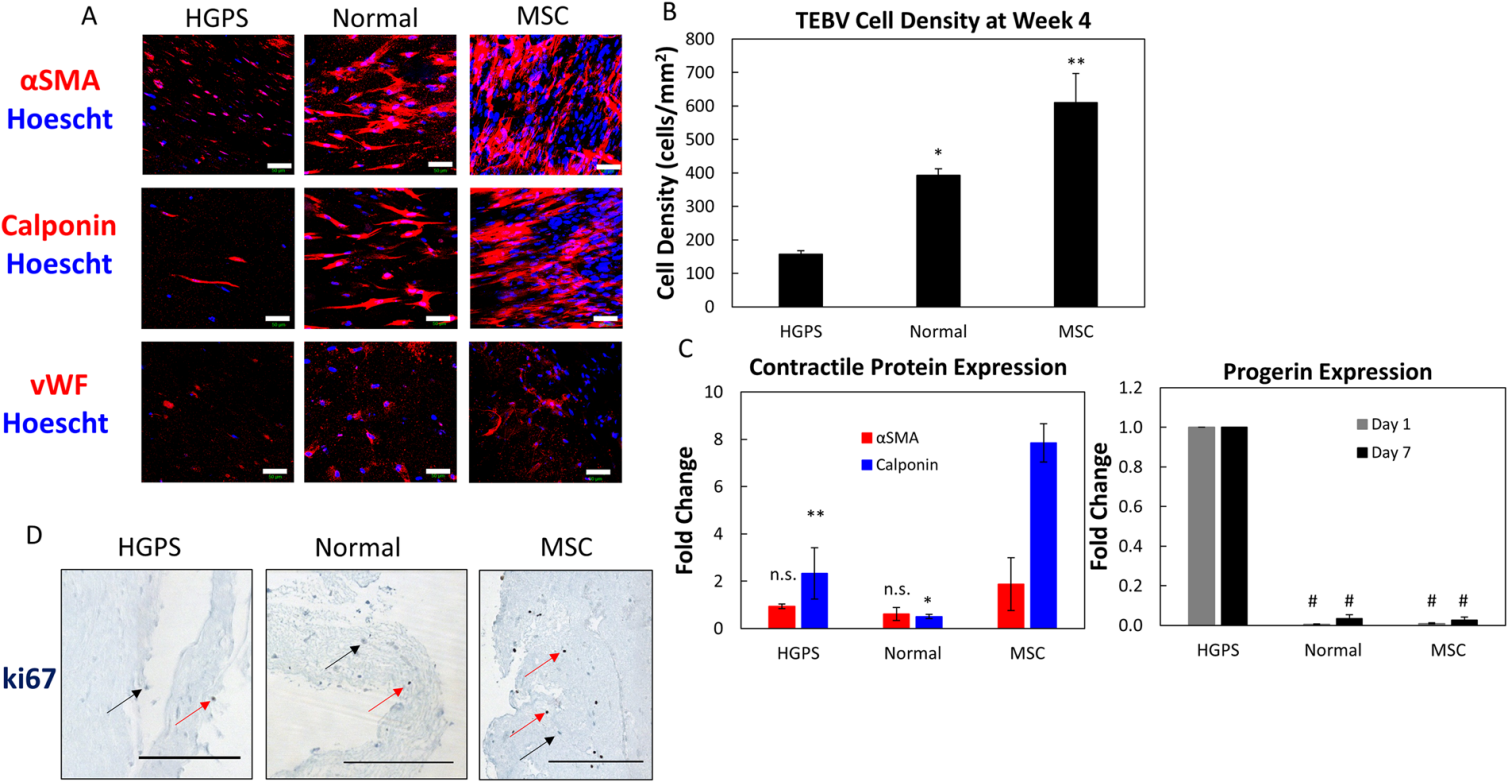
Structural characterization of TEBVs fabricated from MSC or iPSC-derived SMC TEBVs from healthy and Progeria patients. (**A**) Representative images of immunofluorescence staining with α-smooth muscle actin, calponin, and vWF antibodies at week 4 of perfusion on TEBVs fabricated from HGPS iSMCs, normal iSMCs, and MSCs and seeded with hCB-ECs in the lumen. (Scale bar, 50 μm). (**B**) Quantification of cell density from A based on the number of nuclei per field area. (**C**) qRT-PCR of Progerin, alpha-smooth muscle actin and calponin gene expression on MSC, HGPS iSMC and normal iSMC TEBVs. Progerin expression was set at 100% for HGPS iSMC TEBV samples at day 1 and day 7. Alpha-smooth muscle actin and calponin gene expression in TEBVs at day 7 of perfusion culture were normalized to TEBVs on day 1 of perfusion culture. Data normalized to GAPDH expression. (**D**) Histochemical analysis of HGPS iSMC, normal iSMC, and MSC TEBVs at week 1 with ki67. Red arrows indicate ki67 positive cells and black arrows indicate ki67 negative cells (Scale bar, 200 μm). n = 3 TEBVs for each TEBV cell type. *P < 0.05, **P < 0.01, ^#^P < 0.001, n.s. =not significant.

**Figure 4:**
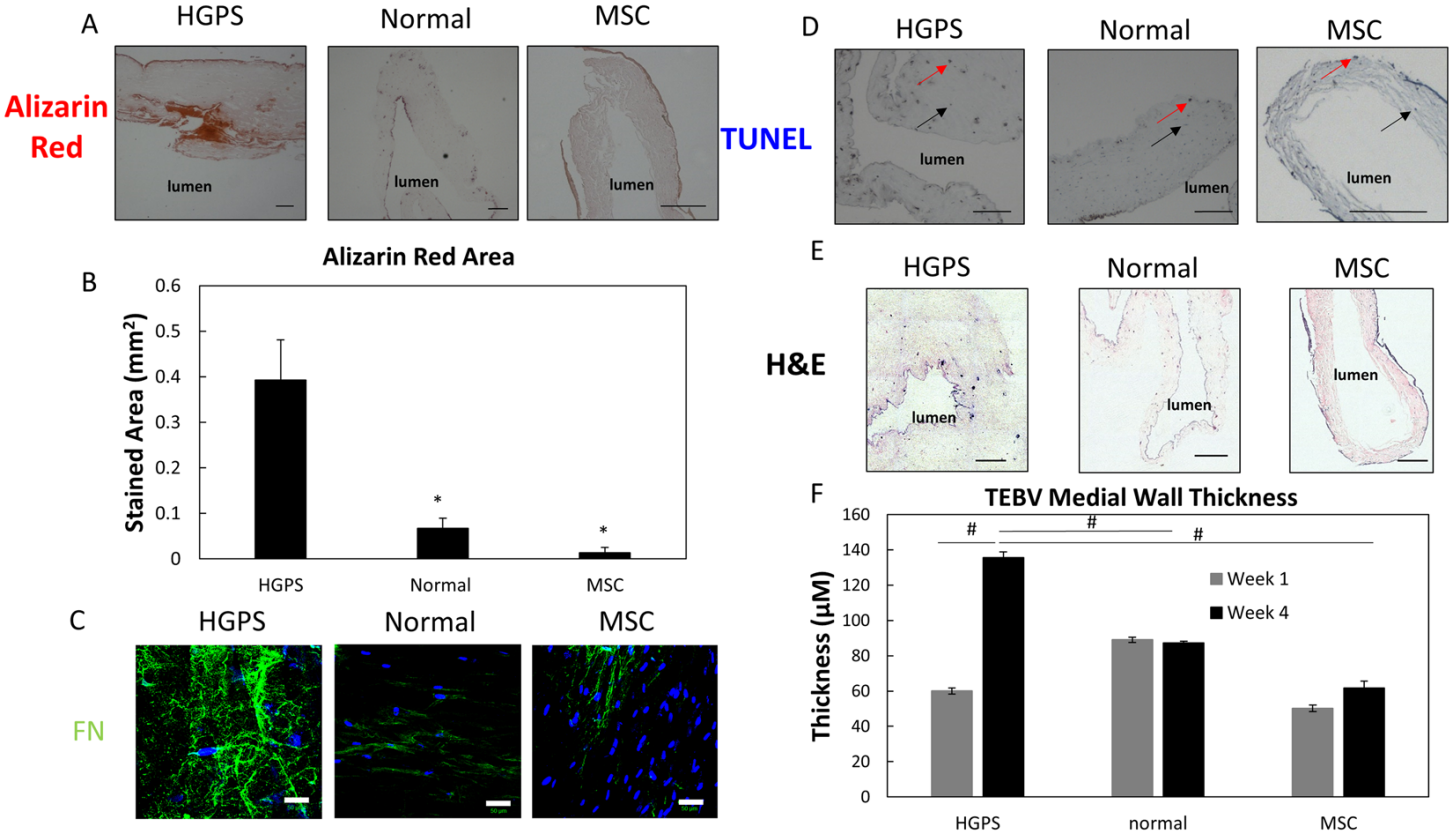
Progeria disease characterization of TEBVs fabricated from MSC or iPSC-derived SMC TEBVs from healthy and Progeria patients. (**A**) Histochemical analysis of HGPS iSMC, normal iSMC, and MSC TEBVs at week 4 with Alizarin Red staining (Scale bar, 200 μm). (**B**) Quantification of A, total area positive for Alizarin Red. (**C**) Representative images of immunofluorescence staining with fibronectin antibodies at week 4 of perfusion on TEBVs fabricated from HGPS iSMCs, normal iSMCs, and MSCs and seeded with hCB-ECs in the lumen (Scale bar, 50 μm). (**D**) Histochemical analysis of MSC, normal iSMC and HGPS iSMC TEBVs at week 4 with TUNEL staining. Red arrows indicate TUNEL positive cells and black arrows indicate TUNEL negative cells (Scale bar, 200 μm). (**E**) Histochemical analysis of HGPS iSMC, normal iSMC, and MSC TEBVs at week 4 with H&E (Scale bar, 200 μm). (**F**) The average thickness of MSC, normal iSMC and HGPS iSMC TEBVs at week 1 and week 4 based on H&E images in E. n = 3 TEBVs for each TEBV cell type. *P < 0.05, **P < 0.01, ^#^P < 0.0001.

To validate the differentiation of the iSMC TEBV platform, we further probed the protein and gene expression of healthy and HGPS TEBVs. The lower functional response to phenylephrine seen in iSMC TEBVs is consistent with the reduced expression of contractile proteins alpha smooth muscle actin (αSMA) and calponin in comparison to MSC TEBVs at week four (Fig. 3A). We also observed a reduction in the expression of the endothelial cell marker vWF in TEBVs fabricated from HGPS TEBVs compared to normal and MSC TEBVs (Fig. 3A). This is consistent with the reduced vasodilation in response to acetylcholine seen in the HGPS TEBVs (Fig. 2C). Immunostaining and hematoxylin and eosin (H&E) staining also indicate lower overall cellularity at four weeks in healthy and HGPS iSMC TEBVs compared to MSC TEBVs (Fig. 3A,B). H&E staining also shows a more crumpled nature of iSMC TEBV constructs in comparison to MSC TEBVs, with HGPS iSMC TEBVs showing the most extensive amount of vessel wall crumpling. This may be due to the decreased mechanical stability of TEBVs fabricated from iPS-derived SMCs compared to MSC TEBVs, which decreases their ability to withstand immunohistochemical processing. The increased extent of this vessel wall crumpling in HGPS iSMC TEBVs may also indicate a more fragile state of these HGPS iSMC TEBVs (Fig. 4E). Quantification of nuclei in immunostained samples shows a significant reduction of cell density in iSMC TEBVs compared to MSC TEBVs, with HGPS iSMC TEBVs having the lowest overall cell density after four weeks of perfusion (Fig. 3B). Immunostaining shows that cell density appears greater at week one compared to week four in both normal and iSMC TEBVs, however, HGPS iSMC TEBVs still show lower cell counts compared to normal iSMC TEBVs even at week one (Fig. 3B and S1). Ki67 staining of TEBV samples after one week of perfusion also shows reduced ki67 positive cells in iSMC TEBVs compared to MSC TEBVs (Fig. 3D). These results suggest increased cell loss in TEBVs fabricated from HGPS iSMCs as well as decreased proliferation potential of iSMCs overall compared to MSCs in our TEBV constructs.

Gene expression analysis by qRT-PCR of TEBVs indicates little or no expression of progerin in MSC and normal iSMC TEBV samples compared to HGPS iSMC TEBVs at day one and after one week of perfusion consistent with reports in 2D cultures (Fig. 3C)^12^. If HGPS samples are normalized to Day 1, there is no change in the progerin expression level over the one week of perfusion (data not shown). LMNA mRNA expression is also increased in normal iSMC TEBVs compared to HGPS iSMC TEBVs at both day one and after one week of perfusion (Fig. S2C). Expression of mRNA for contractile proteins, alpha-smooth muscle actin (αSMA) and calponin, are upregulated after one week of perfusion in MSC TEBVs indicating a maturation of these cells under perfusion culture to a more mature and contractile phenotype as we have previously seen. iSMC TEBVs do not show a significant upregulation of either gene after culture, indicating that they did not undergo further differentiation after fabrication (Fig. 3C). These results are consistent with the improved functional response seen in MSC TEBVs over time versus the consistent functional response seen in iSMC TEBVs.

To estimate the stress in the vessel wall, we applied the Law of Laplace using the flow rate, diameter (Fig. 2A) and thickness measured at 1 and 4 weeks (Fig. 4F). These results indicate that the larger HGPS TEBVs can support a 50% higher stress than the smaller normal and MSC TEBVs. While we cannot directly estimate the elastic modulus, these results suggest that the larger diameters and thicker walls at 4 weeks of the HGPS TEBVs compensates for a weaker vessel modulus.

Supporting this, we observed that HGPS iSMC TEBVs had a higher porosity than the MSC TEBVs. In measuring the pressure drop in the TEBVs, we found significant leakage of fluid across the vessel wall for HGPS iSMC TEBVs. We applied Darcy’s law to estimate the hydraulic permeability^24^. Since we did not measure appreciable flow across Normal iSMC TEBVS or MSC TEBVs, we assumed the fluid flow rate across these vessels was within the measured error of flow rate (0.2 ml/min). With this assumption, we determined that the porosity of the HGPS iSMC TEBVs was at least 68-84 times larger than the values for the Normal iSMC TEBVS or MSC TEBVs. This greater porosity of the HGPS iSMCs suggests that they are mechanically weaker than the Normal iSMC TEBVs and MSC TEBVs and the larger size is a compensatory mechanism to resist the fluid pressure.

We attempted a variety of methods to improve the function of the iSMC TEBVs including mechanical factors, such as pulsatile flow and higher pressures, as well as modulating culture conditions such as addition of doxycycline and ascorbic acid. Perfusion with 10 µg/ml doxycycline had the most profound effect by decreasing the degradation of collagen I and increasing iSMC differentiation in 3D as well as improving vasoactivity of TEBVs made with HGPS iSMCs (Fig. S3). Since doxycycline inhibits matrix metalloproteinase two and nine, these results suggest that the thickening of the vessels is mediated, in part, by matrix metalloproteinases^25, 26^.

### HGPS Disease Characterization of iSMC TEBVs

To determine whether TEBVs fabricated from HGPS iSMCs can develop a disease state *in vitro*, we probed our TEBVs for markers of the HGPS cardiovascular phenotype. Histology of HGPS iSMC TEBVs after four weeks of perfusion culture showed increased amounts of calcification (Alizarin Red, Fig. 4A) and apoptotic cells (TUNEL, Fig. 4D) in comparison to TEBVs fabricated from normal donor cells or MSCs. Quantification of Alizarin Red staining shows significantly increased stained area of Alizarin Red in HGPS TEBVs compared to normal iSMC or MSC TEBVs (Fig. 4B). Histology also shows a significantly thicker medial wall in vessels fabricated from HGPS iSMCs compared to normal iSMC or MSC TEBVs after four weeks of perfusion (Fig. 4E,F). HGPS iSMC TEBVs perfused for one week, however, are not significantly thicker and do not express the same high levels of Alizarin Red or show the same amount of TUNEL positive cells (Fig. S4). HGPS iSMC TEBVs also show increased levels of fibronectin deposition compared to MSC or normal iSMC TEBVs, consistent with previous reports of progeroid cells which exhibited a deregulation of key ECM proteins (Fig. 4C)^27^. HGPS patient fibroblasts have shown increased levels of fibronectin gene expression which could subsequently lead to the vascular stiffening seen in HGPS patient vasculature^7^. These staining results and reduced cell numbers are consistent with previous reports of the HGPS disease phenotype indicating the potential for these vessels to display various HGPS disease characteristics after four weeks of perfusion culture^7, 28^.

### Effect of Rapamycin Treatment on HGPS iSMC TEBVs

**Figure 5:**
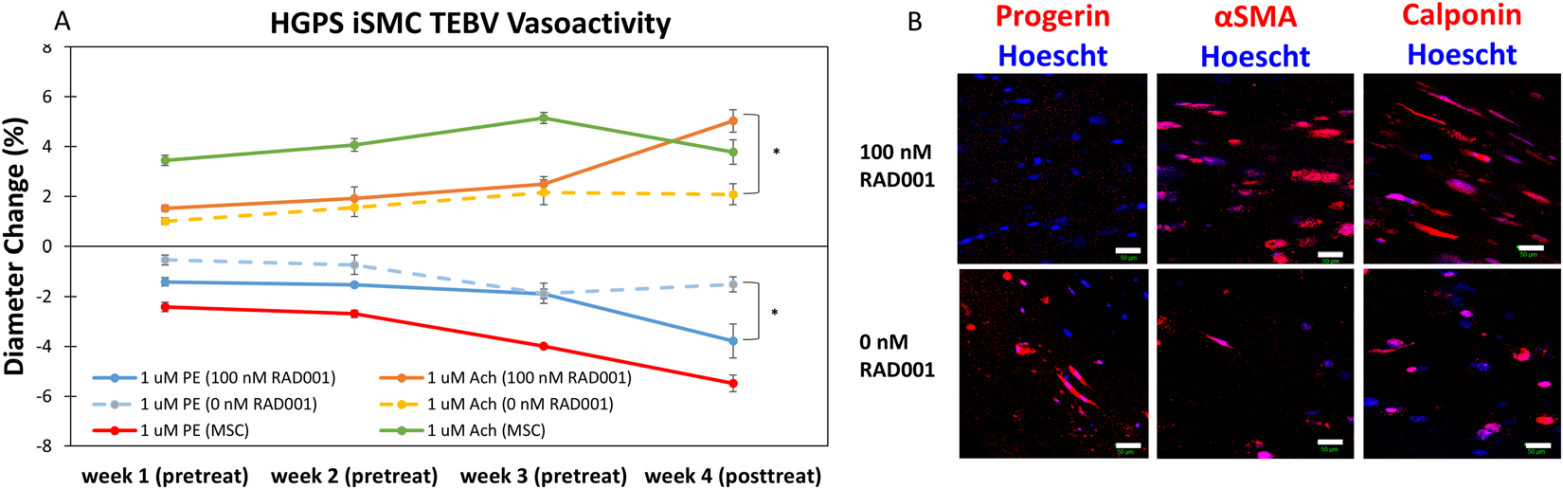
Effect of Everolimus (RAD001) treatment on HGPS iSMC TEBVs structure and function. (**A**) Functional response to 1 μM phenylephrine and 1 μM acetylcholine of HGPS iSMC TEBVs seeded with hCB-ECs after 3 weeks of normal perfusion and 1 week of treatment with 100 nM everolimus (RAD001) compared to untreated MSC TEBVs (From Fig. 2). (**B**) Representative images of immunofluorescence staining with Progerin, α-smooth muscle actin, and calponin on HGPS iSMC TEBVs at week 4 untreated or treated with 100 nM everolimus (Scale bar, 50 μm). n = 3 TEBVs. *P < 0.05.

**Figure 6:**
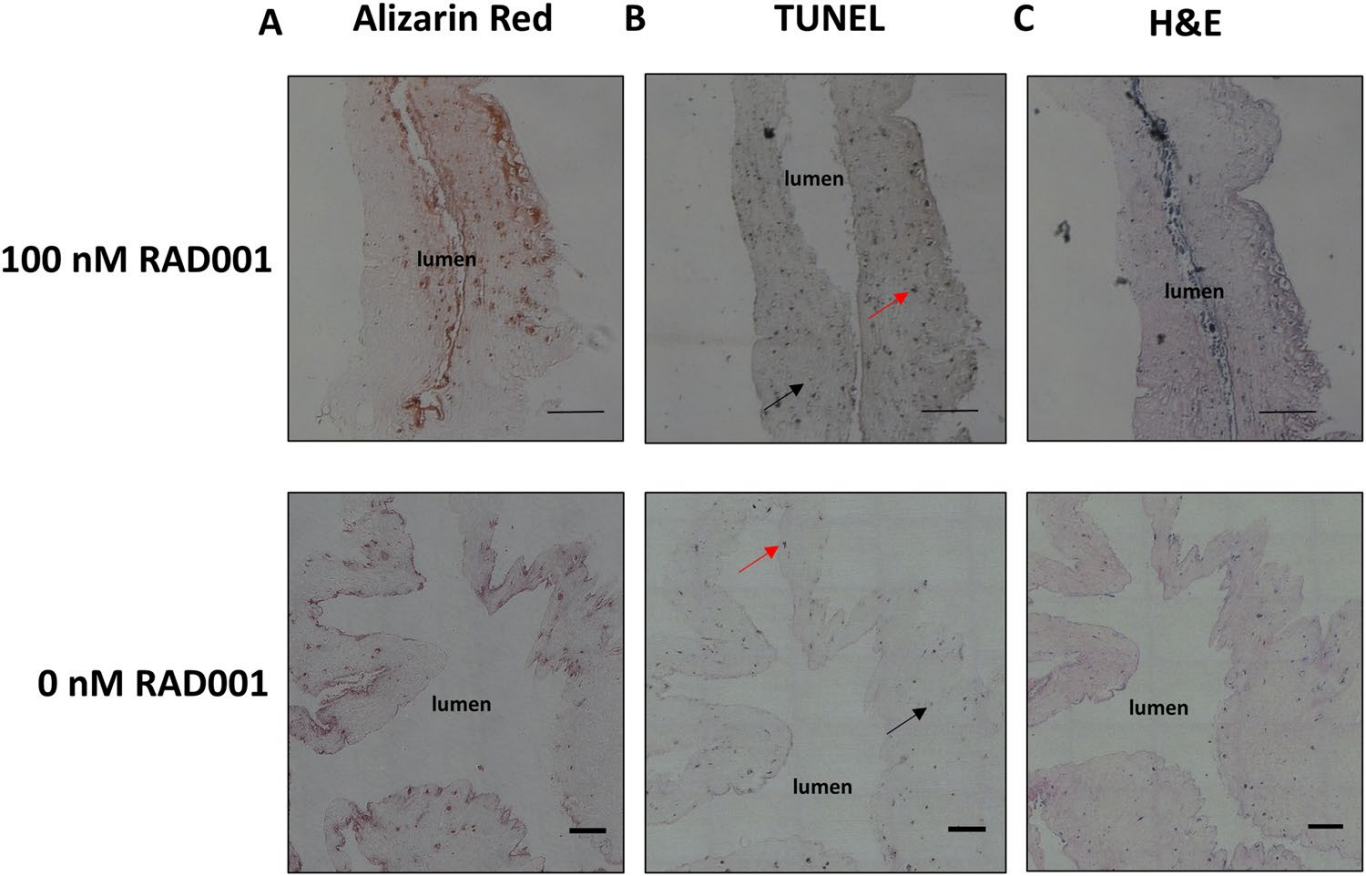
Histochemical analysis of HGPS TEBVs treated with Everolimus vs. untreated controls. (**A**) Alizarin Red stain, (**B**) TUNEL stain and (**C**) H&E stain of HGPS iSMC TEBVs after 3 weeks of normal perfusion and 1 week of treatment with 100 nM RAD001 or HGPS iSMC TEBVs without treatment. Red arrows indicate TUNEL positive cells and black arrows indicate TUNEL negative cells (Scale bar, 200 μm).

Once a disease phenotype was established in HGPS TEBVs, we tested the effects of the rapamycin analog RAD001 (Everolimus) on the functional capacity of TEBVs fabricated from HGPS iSMC TEBVs. TEBVs were fabricated using HGPS iSMCs and hCB-ECs and perfused for three weeks to develop disease attributes within the TEBVs. At week three, 100 nM of RAD001 was added to the culture media and perfusion continued for one week with the media being changed twice during the one week period. In comparison to untreated controls, constriction in response to 1 μM phenylephrine and dilation in response to 1 μM acetylcholine increased significantly in HGPS iSMC TEBVs treated with 100 nM RAD001 for one week (Fig. 5A). This increased functional response correlated with increased expression of the contractile proteins αSMA and calponin (Fig. 5B) indicating the ability to improve vessel function and iSMC maturation with HGPS therapeutics. When compared to untreated MSC TEBVs prior to week four, HGPS iSMC TEBVs treated with 100 nM RAD001 for one week respond at comparable levels to 1 μM phenylephrine and 1 μM acetylcholine. Interestingly, histology at week four post-treatment with Everolimus did not show a reduction in calcification or apoptosis as indicated by histological staining for Alizarin Red and TUNEL, respectively (Fig. 6). This indicates that these processes were already underway and further treatment or altered drug concentrations may be needed to reverse the developed disease state. Most importantly though, immunostaining did show a reduction in expression of progerin and restoration of the normal nuclear shape (Fig. 5B). This is consistent with previous reports in 2D that indicate the ability of rapamycin to help solubilize progerin and clear it through autophagy^29, 30^.

## Discussion

The development of an *in vitro* 3D blood vessel model that recapitulates the biological structure and function of those found *in vivo* is of great interest for modeling HGPS and other rare diseases affecting the cardiovascular system^31^. A limited number of animal models are available for HGPS and currently only two exist that recapitulate the symptoms of the HGPS cardiovascular phenotype (the BAC G608G model and the G609G knock-in model)^28, 32, 33^. Despite displaying many of the symptoms associated with the LMNA mutation, neither model develops fibrotic atherosclerotic lesions, which is the primary cause of death in HGPS. This indicates a key limitation of these models for drug screening purposes. The need still exists, however, to discover therapeutics and conduct clinical trials even with limited patient populations^34^. In order to overcome these limited test populations, personalized medicine efforts have focused on creating patient-specific *in vitro* models using iPS-derived cell sources in order to tailor drug testing efforts to that particular patient^35, 36^. This would allow for therapeutic discovery for a wide variety of diseases, both common and rare. In conjunction with animal models, this could increase accuracy of drug screening prior to clinical trials.

Our studies show that we can produce functional, tissue engineered blood vessels from iPSC-derived patient cells from both healthy and HGPS donor cells and perfuse them at physiological flow rates for multi-week time periods. Dash *et al*. previously created a functional disease model from iPS-derived SMCs in 3D vascular rings that display a cardiovascular disease phenotype^37^. These SMCs were differentiated and exhibited contractile activity. Our work, however, demonstrates endothelial-mediated vasodilation of the iPS-derived healthy and HGPS SMCs and assesses pathology using cells from an individual with progeria. This model, therefore, shows the potential to examine patient specific variability to various therapeutics by utilizing the assays that we have validated (vasoactivity, protein characterization, pathology, etc.) to probe for variations in patient response since iPSCs allow for the conservation of genetic background post-differentiation. Although TEBVs fabricated from iSMCs of a healthy individual did not show the same level of function or cell proliferation and maturation as TEBVs produced from primary MSC cultures over long term culture periods, they can respond to known vascular stimulants and maintain these responses at a stable level for up to a month.

A known challenge with iPSCs is that the resulting cell sources do not always display a mature phenotype or comparable function to primary cell sources^38^. This reduced function and decreased proliferation could explain why MSC TEBVs show stronger responses to vasoagonists that significantly increase as the MSCs differentiate and mature. Also under perfusion conditions and in the presence of ascorbic acid, MSCs proliferate and differentiate into a very contractile state^5, 39^. Our current conditions may not induce the same level of contractile state or proliferative potential for the iSMCs to allow them to show such strong functional responses as MSCs even though normal iSMC TEBVs and MSC TEBVs show no significant functional differences at week one. Importantly, our studies show a reduction in function from TEBVs fabricated from HGPS iSMCs in comparison to normal iSMC control TEBVs, although the difference is not significant at later time points when disease pathology has developed. Therefore, future work involving the improvement of the functional capabilities of these cells in TEBVs may show a more significant difference in the function of healthy and diseased donor cells in TEBVs. This increased function will be necessary to enhance the sensitivity of our system as a disease model and as a drug testing platform for future therapeutic analysis. Nonetheless, the stable response seen in these iSMC TEBVs can be useful for monitoring any changes that occur in response to drug candidates introduced into the system since changes in response to drugs can be assumed to be due to the drug compound itself rather than cell or TEBV construct changes. Other cell sources previously implemented in our TEBV system, such as MSCs and fibroblasts, have not shown such stable responses over long term perfusion culture, which is likely due to progressive maturation of these cells into a contractile state^20^. Therefore, iSMCs may be an advantageous cell source in TEBVs for drug modeling and disease testing based on their ability to carry a specific disease phenotype and maintain consistent structure and function over long-term culture periods if we can induce the same level of contractility in the iSMC TEBVs that we have seen at later time points in the MSC TEBVs.

Our model also shows key differences in structure, function and tissue pathology between TEBVs fabricated from healthy and HGPS cells suggesting the ability to model a human disease state in an *in vitro* 3D MPS platform. In particular, we observed increased calcification, decreased cellularity, and reduced vasoactivity which are known aspects of the HGPS phenotype^40^. We also observed an increase in medial wall thickening over the four-week time course of perfusion in TEBVs fabricated from HGPS iSMCs, which was not seen in healthy iSMC TEBVs or MSC TEBVs. A biomechanical analysis suggests that this wall thickening by secretion of the extracellular matrix may serve as a compensatory mechanism for the weaker vessel wall. Although the exact mechanism is not known as to how this occurs *in vivo*, mouse models of the HGPS cardiovascular phenotype display similar structural changes despite the progressive cellular loss in the vasculature^11, 28^. Due to the increase in extracellular matrix proteins in HGPS iSMC TEBVs, we hypothesize that this excessive ECM deposition is the potential cause of the medial wall thickening we have observed, however, further studies will be necessary to validate this hypothesis. We also observed a reduction in endothelial cell presence and reduced acetylcholine response in TEBVs fabricated from HGPS iSMCs. Although little is known about the effects of HGPS on the endothelium, previous reports have indicated that endothelial cells with Lamin A defects showed premature senescence and increased inflammatory response^41, 42^. Our current system utilizes hCB-ECs from a healthy donor, however, incubating healthy ECs with conditioned media from ECs overexpressing progerin can induce similar dysfunction^43^. Therefore, our system may indicate the effect of dysfunctional Progeria SMCs on the endothelium that could not have been observed in 2D monocultures of either cell type. Since our intention with this model was to isolate the effects of iSMCs on TEBV structural development and functional response, we chose to use hCB-ECs from a healthy donor unrelated to either iSMC donor. To fully understand the effects of HGPS on the endothelium, however, future work will involve incorporating iPSC-derived ECs from the same donor as the iPSC-derived SMCs into the TEBV constructs to create a more relevant personalized medicine platform for disease studies and drug testing.

The rapamycin analog, Everolimus, has recently been proposed as a treatment for HGPS patients, however, previous reports on this compound have only been tested in 2D cell cultures^29, 30^ and did not evaluate functional responses. A recent clinical trial to test the utility of this compound was implemented in April 2016, however, the limited patient pool creates a bottleneck in determining the safety and efficacy of this trial. Phase I studies only involve slowly and incrementally testing increasing dosages of the drug on three patients at a time and then observing the effects to determine the safest maximal dose (SMD). Phase II will then involve the co-treatment of Everolimus and the farnesyltransferase inhibitor Lonafarnib, which has previously been shown to increase HGPS patient lifespan and reduce the cardiovascular defects associated with the disease^44, 45^. This trial is predicted to take 3.5-4 years and cost around $2.5 million dollars. The small test group size may not be able to accurately predict wide spread use of the drug if it receives FDA approval and eventually reaches the market. Our TEBV system has the potential to expedite this testing process and create a platform that can be used to test the specific effects of the drug on any patient. Although human clinical trials will still be necessary for verifying drug efficacy prior to FDA approval, an *in vitro* platform involving human cells that replicates a 3D tissue structure will help bridge the gap between 2D cell culture studies and *in vivo* animal models during preclinical trials. By better replicating the microenvironment of human vasculature, a TEBV model of HGPS and other cardiovascular related diseases will aid in verifying results seen in other preclinical models and provide improved confidence in drug efficacy and toxicity for clinical testing.

Treatment with Everolimus on TEBVs fabricated from cells of patients with HGPS show improvement in function as well as increased expression of smooth muscle contractile proteins. This may indicate that the removal of progerin by rapamycin restores the function of the HGPS smooth muscle cells. Since dysfunction and loss of smooth muscle cells in the vasculature of HGPS patients is a potential cause of many of the atherosclerotic symptoms known to the HGPS phenotype such as calcification and fibrosis, these results could indicate the potential of rapamycin to reverse this cellular malfunction. We did see a reduction in the expression of the progerin protein in TEBVs treated with RAD001 as well as an increase in overall cellularity. This could indicate that the functional improvement we see in the TEBVs is due to this decrease in progerin toxicity and suggests the need for further study into the mechanism with which this functional improvement occurs in the 3D engineered tissue.

Although we did see improvement in TEBV function after exposure to rapamycin the presence of calcification was not ameliorated by this treatment (Fig. 6). This could indicate the need to treat diseased TEBVs for longer time periods to remedy disease pathology that has already developed within the vasculature. Another possibility is that rapamycin may be necessary as a pre-emptive measure to prevent the development of cardiovascular disease symptoms before they occur. If the main benefit of rapamycin is the ability to solubilize progerin and improve SMC function, rather than to reduce the vascular defects that develop from the disease state, it may be necessary to apply the drug early on to prevent the development of the disease symptoms in the first place. Future studies will be necessary to parse out these details and determine how rapamycin can effectively be used to treat the symptoms of HGPS and potentially treat those at risk for cardiovascular disease. In either case, our system has shown the capability to provide extended insight into this drug’s efficacy compared to previous 2D and animal studies in an expedited manner.

Since little patient data is available on how cardiovascular disease develops in HGPS, it has been hard to make clear conclusions on the connection between normal cardiovascular aging and HGPS. Although it is currently proposed that the two disease states may be related, there are still key differences that lead to uncertainty about directly relating the two conditions. For example, it has been observed that there is increased fibrosis and less lipid accumulation in HGPS patient vasculature compared to those suffering from normal cardiovascular disease, which makes it difficult to definitively relate the two disease states for drug development purposes^27^. Therefore, by creating a platform that can develop a vascular disease phenotype may allow future studies to compare the progression of both disease states *in vitro*.

Another key advantage of our system is the rate at which we can develop these disease characteristics within our TEBVs fabricated from human HGPS cell sources. Samples at week one do not show significant disease pathology (Fig. 6), however, by week four many disease characteristics were evident, including reduced cell density, medial wall thickness, calcification and apoptosis. This four-week development of cardiovascular disease pathology in TEBVs with cells from HGPS patients is greatly accelerated compared to mouse models of HGPS, which can take up to 12 months to develop relevant HGPS cardiovascular symptoms. The cost of running our system is also much less than mouse models and we can non-destructively monitor the vessel function at any time during the study. In addition, using human cells sources provides a more relevant understanding of how HGPS progresses *in vivo* in humans and could help elucidate further mechanisms controlling HGPS cardiovascular disease development.

There are, however, still limitations to this model due to its structural simplicity and isolation from other tissue types. In particular, this TEBV model does not develop all the pathologies associated with cardiovascular disease seen in larger arteries including lipid accumulation which has previously been associated with the HGPS phenotype^7, 16^. This aspect of cardiovascular disease has been difficult to replicate in previous *in vitro* TEBV models as well and should be further optimized in order to fully recapitulate a cardiovascular disease state *in vitro*. Nonetheless, the ability of our system to develop multiple disease characteristics that can be studied for drug efficacy shows its potential as a useful drug testing platform.

This study shows that our iSMC TEBV platform has the potential to predict functional and pathological disease characteristics that may allow for the development of more efficacious drugs to treat HGPS. Our model may also be able to extend to other disease states including normal cardiovascular aging to expedite pre-clinical studies and reduce drug toxicity in the general patient population.

## Materials and Methods

### Cell isolation and culture

hCB-ECs were derived from human umbilical cord blood obtained from the Carolina Cord Blood Bank, and all patient identifiers were removed prior to receipt. Isolation and culture protocols for hCB-ECs were approved by the Duke University Institutional Review Board and hCB-ECs were derived as previously described^46^. hCB-ECs were cultured in EBM-2 (Lonza, Basel, CH) supplemented with EGM-2 Single Quots Kit (Lonza), 1% Pen/Strep (Gibco Life Technologies, Grand Island, NY), and 10% fetal bovine serum (HI-FBS, Gibco). Media was changed every two days. hCB-ECs at passage 5-8 were used for all experiments.

Human bone-marrow derived MSCs were provided by Darwin J. Prockop of Texas A&M Institute for Regenerative Medicine^47^. hMSCs were cultured in MSC medium (α-minimum essential medium (Gibco) supplemented with 20% fetal bovine serum (Gibco), 1% l-glutamine (ThermoFisher) and 1% Pen/Strep (Life Technologies). Media was changed every two days. MSCs were used between passage 5 and passage 8.

Primary skin fibroblasts with the classic G608G HGPS mutation (HGADFN167) and control primary skin fibroblasts (HGFDFN168) were provided by the Progeria Research Foundation and converted to iPSCs as previously described^48^. iSMCs were generated from iPSCs as previously described^19, 21^. Briefly, pre-SMCs were plated on Matrigel (BD) coated plates and cultured to 90% confluence in SMGM (Lonza) for one week. Pre-SMCs were trypsinized with TrypLE Express (Life Sciences) and passaged 1:2 onto gelatin coated plates in DMEM supplemented with 5% FBS (Gibco) to induce differentiation. Cells were cultured for four days and then serum levels were lowered to 1% for three more days. iSMCs were passaged 1:2 with trypLE Express onto gelatin-coated plates and maintained in iSMC medium (DMEM supplemented with 5% FBS (Gibco) and 1% Pen/Strep (Life Technologies). iSMCs were passaged 1:2 when 90% confluence was reached. Media was changed every two days. All iSMCs were used after passage 1 and normal iSMCs were not used above passage 3 while HGPS iSMCs were not used above passage 5.

#### TEBV fabrication and functional testing

TEBVs were fabricated as previously described^20, 49^. Briefly, 1.5 × 10^6^ MSCs, normal iSMCs, or HGPS iSMCs were dissociated and resuspended in 300 uL of respective media (MSC or iSMC) and embedded in a 2.05 mg/mL rat tail collagen type 1 solution (Corning, Corning, NY) in 0.6% acetic acid. Serum-free 10x DMEM was added at a 1:10 ratio to the collagen solution. The pH of the solution was raised to 8.5 by the addition of 5 M NaOH, allowing the solution to gel. Prior to gelation, the solution was placed in a 3 mL syringe mold (BD Biosciences, San Jose, CA) with the pump removed and a two-way luer-lock stopcock attached. An 800 μm diameter steel mandrel was inserted in the middle and held in the center with parafilm wrapped over the syringe opening. The solution was allowed to gel for 30 minutes at room temperature. After gelation, the vessel construct was transferred onto 0.2 μm nylon filter paper (Whatman, Maidstone, UK) on 10 KimWipes. Plastic compression was applied to the construct by suspension in the filter paper for seven minutes to remove water. Once compressed, the TEBVs were placed in custom chambers and sutured onto grips at both ends.

TEBVs were endothelialized by detaching adherent hCB-ECs with 0.05% Trypsin/EDTA (Lonza) and resuspending them at 0.5 × 10^6^ cells in 0.5 mL EGM2. hCB-ECs were perfused through the lumen using 1 mL slip-tip syringes (BD) connected to the grips of the custom chambers. The TEBVs were evenly endothelialized by rotating the chambers at 10 revolutions per hour for 30 minutes at 37 °C on a rotation platform. Chambers were attached to a flow circuit containing a media reservoir connected by tubing. Continuous, steady, laminar flow at a flow rate of 2 mL/min was applied to the vessels by attaching the perfusion circuit to a peristaltic pump in order to apply a physiological shear stress of 6.8 dynes/cm^2^ to the TEBVs (Masterflex, Gelsenkirchen, DE). The TEBVs were matured for a maximum of four weeks and media was changed three times per week. HGPS and normal iSMC TEBVs were perfused in iSMC medium. MSC TEBVs were perfused in MSC medium supplemented with 82.5 μg/mL L-Ascorbic Acid (Sigma, St. Louis, MO) for the first week of perfusion to induce differentiation to the SMC phenotype and then MSC medium for the following weeks of perfusion.

Vessel vasoactivity was measured as the change in diameter of the TEBVS after exposure to 1 μM phenylephrine (Sigma) and 1 μM acetylcholine (Sigma). Vasoactivity was measured in the same perfusion circuit that TEBVs were cultured in and imaged using a stereoscope (AmScope) while being recorded with ISCapture software. 1 μM phenylephrine was added to a syringe port (Ibidi) integrated in the flow circuit and after 5 minutes 1 μM acetylcholine was added. This process was performed weekly for month long studies. Screen shots were taken at 30 seconds (prior to phenylephrine addition), at five minutes (after phenylephrine addition) and at 10 minutes (after acetylcholine addition). The diameter at each time point was determined by averaging four random widths along the length of the vessel using ImageJ. Vessel diameter measurements were defined at the 30 second time point. Vasoconstriction in response to phenylephrine was calculated as the percent change in diameter from the initial diameter at 30 seconds before addition of phenylephrine to the diameter at 5 minutes after addition of phenylephrine. The vasodilation in response to acetylcholine was calculated as the percent change in diameter from the constricted state at 5 minutes to the diameter at 10 minutes.

#### Immunofluorescence staining

TEBVs were fixed in 10% formalin for 10 minutes attached to the chamber grips to maintain structural integrity. TEBVs were then placed in a well-plate and fixed in 10% formalin for an additional 50 minutes for a total of one hour of fixation. TEBVs were washed with DPBS two times and then cut en face to expose the lumen. Sections stained with intracellular proteins (αSMA, calponin, and vWF) were permeabilized with 0.1% Triton-X for 5 minutes and then washed three times with DPBS. All samples were blocked in 10% goat serum in DPBS for 8 hours at room temperature. Samples were incubated with primary antibody at 1:100 in 10% goat serum overnight at 4 °C. Samples were washed three times with DPBS and then incubated with secondary antibody at 1:500 in 10% goat serum for one hour at room temperature. Samples were washed three times and incubated with Hoescht 33342 at 1:1000 in DPBS for 5 minutes at room temperature. Samples were washed three times with DPBS and mounted onto glass slides with Fluor Save (Calbiochem, Billerica, MA). TEBVs were imaged using a Zeiss 510 inverted confocal microscope at 20x magnification and analyzed using Ziess LSM image browser. Primary antibodies used were rabbit anti-calponin (Abcam, Cambridge, MA), rabbit anti-αSMA (Abcam), rabbit anti-vWF (Abcam), rabbit anti-laminin (Abcam), mouse anti-fibronectin (Abcam), rabbit anti-collagen IV (Abcam), goat anti-lamin A/C (Santa Cruz, Dallas, TX) and rabbit anti-progerin^30^. Secondary antibodies used were Alexa Fluor 594 goat anti-rabbit, Alexa Fluor donkey anti-goat and Alexa Fluor 488 goat anti-mouse (Life Technologies). Each condition was imaged by immunostaining with 3-4 TEBVs.

For 2D iSMC cultures, cells were fixed 4% paraformaldehyde for 20 min and then washed three times with DPBS. Cells were blocked in 4% BSA in DPBS for one hour at room temperature. Cells were incubated with primary antibody at 1:250 in 4% BSA overnight at 4 °C. Cells were then washed with DPBS three times and then incubated with secondary antibody at 1:500 in 4% BSA for one hour at room temperature. Cells were washed three times with DPBS and incubated with Hoescht 33342 at 1:1000 in DPBS for 5 minutes at room temperature. Samples were washed three times with DPBS and imaged on a Nikon Eclipse TE2000-U microscope at 10x magnification using the NIS Elements software and analyzed using ImageJ.

#### RT-Quantitative PCR and Primers

Total RNA was extracted from MSC, normal and HGPS iSMC TEBVs using the Aurum Total RNA Mini Kit (BioRad, Hercules, CA). TEBV medial cells and hCB-ECs were not separated during RNA extraction in order to reduce medial cell loss and increase RNA yield. Genes of interest were only associated with medial cells. RNA was reverse transcribed into cDNA using the iScript cDNA Synthesis Kit (Bio-Rad). RT-PCR was performed in triplicate using the iQ SYBR Green Supermix (Bio-Rad) and the CFX Connect Real-Time PCR Detection System (Bio-Rad). Primers for amplifying Calponin and α-smooth muscle actin were previously described^20^. As an internal control, human GAPDH was purchased from real-time primers (VHPS-3541, Elkins Park, PA). Primer sequences for amplifying human progerin and Lamin A/C were previously described^19^.

#### Histology Imaging and Analysis

TEBVs were fixed in 10% formalin for 10 minutes attached to the chamber grips to maintain structural integrity. TEBVs were then placed in a well-plate and fixed in 10% formalin for an additional 50 minutes for a total of one hour of fixation. TEBVs were preserved in 70% ethanol until paraffin embedded. Paraffin embedded cross-sections were then stained with hematoxylin/eosin, Alizarin Red, ki67 and TUNEL. Human bone was used as a positive Alizarin Red control, human tonsil was used as a positive ki67 control and mouse liver was used as a positive TUNEL control. Images were taken on a Nikon Eclipse TE2000-U microscope at 20x magnification using the NIS Elements software and analyzed using ImageJ.

TEBV thickness was measured by averaging the distance from the outer vessel wall to the inner lumen at 15 random points on histology cross-sections imaged at the same magnification. Alizarin Red was quantified as the total area stained positive for Alizarin Red while maintaining the same threshold value between all samples. Three different TEBV cross-sections were analyzed per cell type at each time point for each measurement. Cell density was determined by the number of nuclei per field area in three separate immunostaining images for each of three independent samples of each TEBV cell type.

#### Live/Dead staining

TEBVs were incubated in iSMC media containing 2 μM Calcein AM and 1 μM Ethidium homodimer-1 (Life Technologies) for 1 hour at 37 C. TEBVs were then mounted on a slide with DPBS and imaged using a Zeiss 510 inverted confocal microscope at 20x magnification and analyzed using Ziess LSM image browser.

#### Statistical Analysis

Statistical analysis was performed using JMP 13 Pro (SAS). Data was analyzed using a one-way or two-way ANOVA. Post-hoc Tukey’s HSD was used to compare means and repeated measures ANOVA was performed for all time-dependent measures. All data is represented as mean ± S.E.M. and p < .05 was considered significant.

### Data Availability

The datasets generated during and/or analysed during the current study are available from the corresponding author on reasonable request.

## Electronic supplementary material

Supplementary Figures
