# Supplementary material for: A Tissue Engineered Blood Vessel Model of Hutchinson-Gilford Progeria Syndrome Using Human iPSC-derived Smooth Muscle Cells
Source: Sci Rep. 2017 Aug 15;7:8168. doi: 10.1038/s41598-017-08632-4 (PMC5557922; doi:10.1038/s41598-017-08632-4)
Supplement: Supplementary file 1 — Supplementary Figures [file 41598_2017_8632_MOESM1_ESM.pdf]

TITLE

A Tissue Engineered Blood Vessel Model of Hutchinson-Gilford Progeria Syndrome Using Human iPSC-derived Smooth Muscle Cells

AUTHORS AND AFFILIATIONS

Leigh Atchison[1], Haoyue Zhang[2], Kan Cao[2], George A. Truskey[1*]

[1]Department of Biomedical Engineering at Duke University, Durham, NC 27708

[2]Department of Cell Biology and Molecular Genetics at University of Maryland, College Park, MD 20742

CORRESPONDING AUTHOR

Dr. George A. Truskey (919-660-5147, george.truskey@duke.edu) R. Eugene and Susie E. Goodson Professor of Biomedical Engineering Duke University, Durham, NC 27708

SUPPLEMENTARY FIGURES

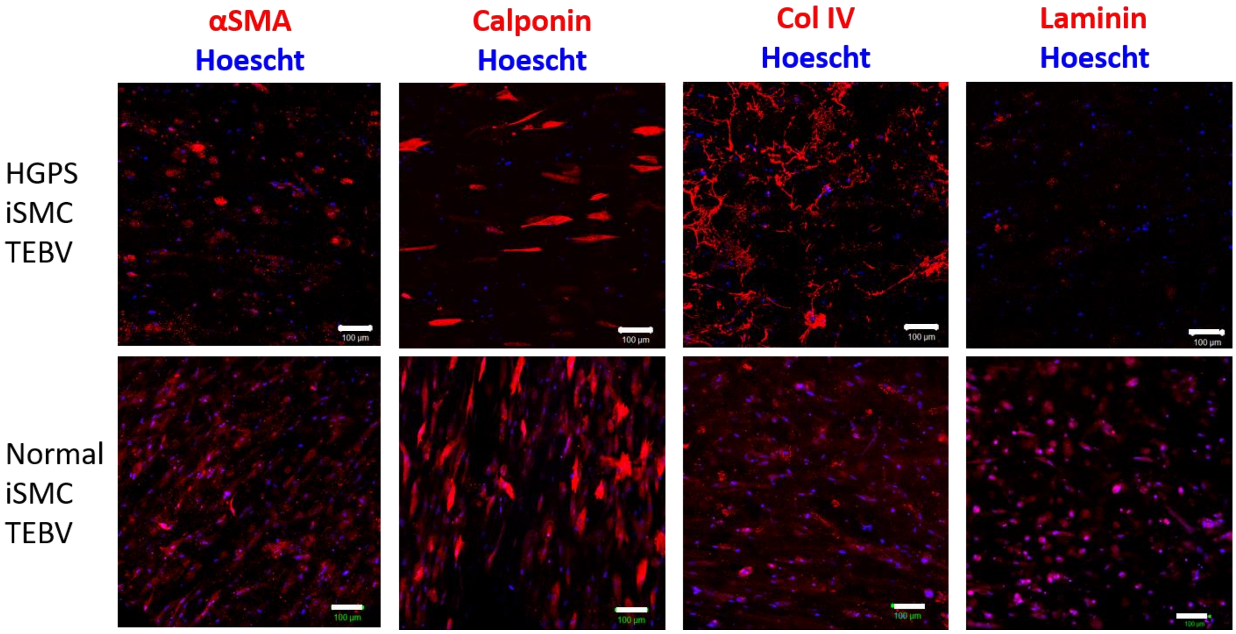

Figure S1: Protein characterization of iSMC TEBVs at Day 7. Representative images of immunofluorescent stains with (A) anti-alpha smooth muscle actin, (B) calponin, (C) collagen IV and (D) laminin on both normal and HGPS iSMC TEBVs after 1 week of perfusion culture (Scale bar, 100 μm)

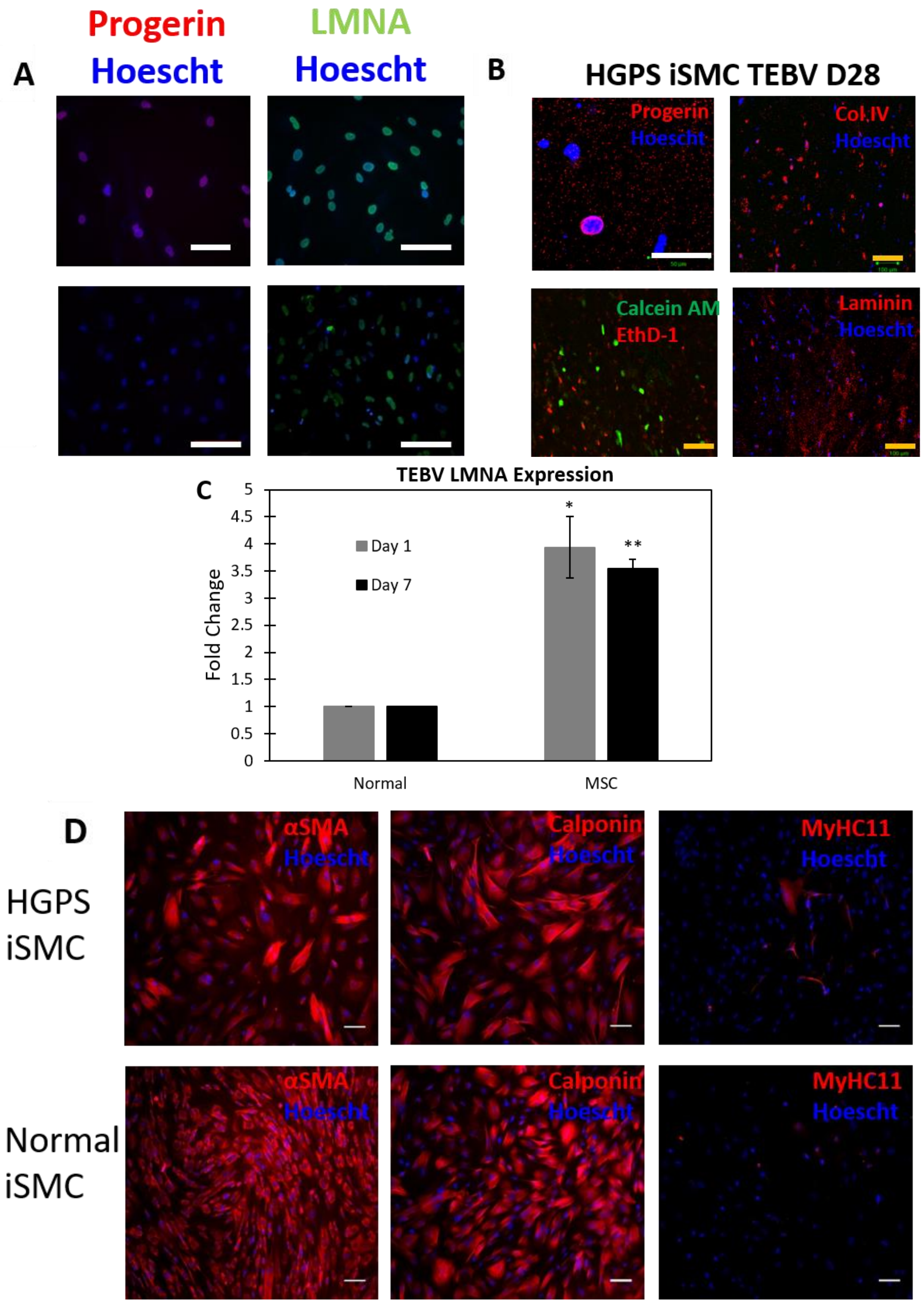

Figure S2: (A) Representative immunofluorescent images with anti-lamin A/C and progerin antibodies of normal and HGPS iSMC after 14 days in 2D culture (Scale bar, 100 μm). (B) Representative immunofluorescent images of HGPS iSMC TEBVs after 28 days of perfusion with anti-progerin, anti-collagen IV, and anti-laminin antibodies and Calcein AM and EthD-1 (white scale bar = 50 μm, yellow scale bar = 100 μm). (C) qRT-PCR of Lamin A/C gene expression on HGPS and normal iSMC TEBVs at D1 and D7 of perfusion culture normalized to HGPS iSMC TEBVs; n=3 TEBVs, *P<.05, **P<.01. (D) Representative immunofluorescent images in 2D culture, prior to formation of TEBVs, of HGPS and normal iSMCs with anti-alpha smooth muscle actin, anti-calponin and anti-smooth muscle myosin heavy chain 11 (scale bar= 100 μm).

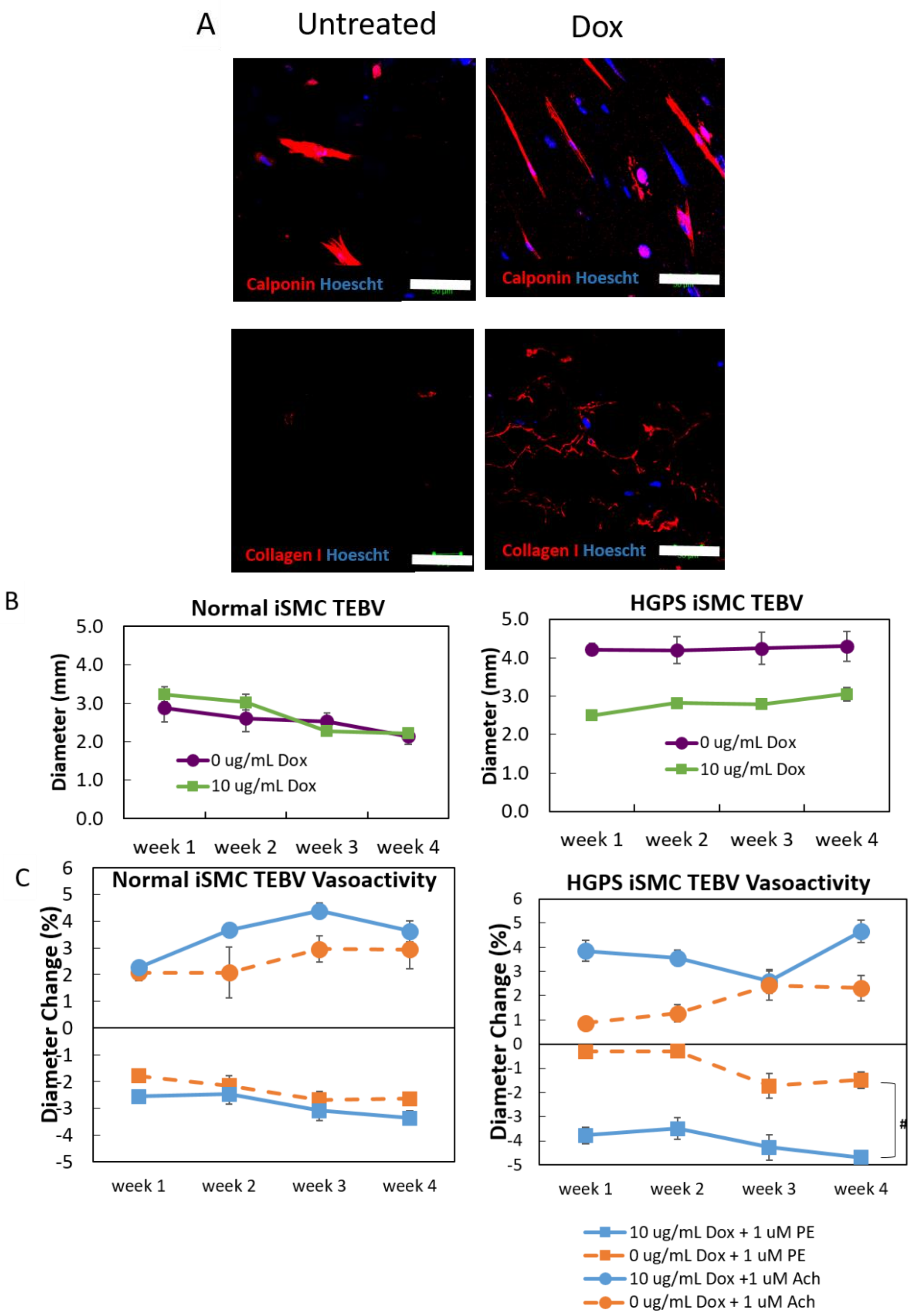

Figure S3: (A) Representative immunofluorescent images of normal iSMC TEBVs after four weeks of perfusion with 10 µg/mL doxycycline or untreated and stained with anti-calponin and anti-collagen I (scale bar=100 µm). (B)Weekly outer diameter measurements of normal iSMC TEBVs seeded with hCB-EPCs or HGPS iSMC TEBVs seeded with hCB-EPCs for four weeks. (C) Weekly vasoactive response to 1 µM phenylephrine or 1 µM acetycholine of normal iSMC TEBVs seeded with hCB-EPCs or HGPS iSMC TEBVs seeded with hCB-EPCs for four weeks. n=3 TEBVs, #P<.0001 at each week.

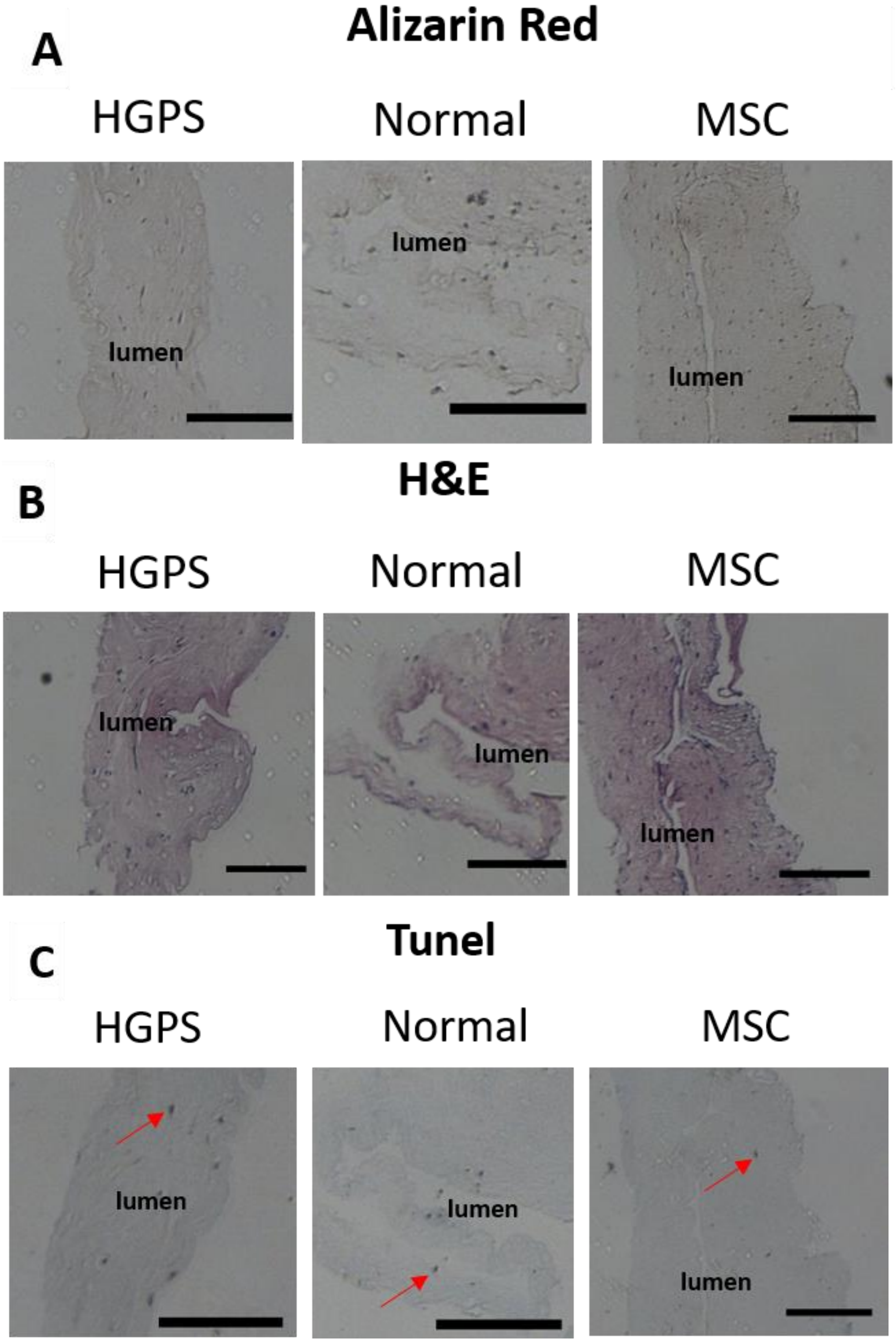

Figure S4: Histochemical analysis of iSMC or MSC TEBVs at Day 7. (A) Alizarin Red stain (B) H&E stain and (C) Tunel stain of HGPS iSMC, normal iSMC or MSC TEBVs after 1 week of perfusion culture (Scale bar, 200 μm).

Cell Type	***Source***	***Cell Line***	***Donor Age***	***Passage Range***
MSC	***Texas A&M Institute for Regenerative Medicine***	***N/A***	***unknown***	***p5-p8***
Normal iSMC	***Progeria Research Foundation***	***HGADFN167***	***8 yrs 5 months***	***p1-p3***
HGPS iSMC	***Progeria Research Foundation***	***HGFDFN168***	***40 yrs 5 months***	***p1-p5***
hCB-EPCs	***Carolina Cord Blood Bank***	***N/A***	***newborn***	***p5-p8***

Table S1: TEBV cell sources
